# Immunogenicity and reactogenicity of inactivated SARS-CoV-2 vaccines in healthy adults

**DOI:** 10.3389/fimmu.2023.1152899

**Published:** 2023-07-25

**Authors:** Yufei Wu, Ping Huang, Mingjie Xu, Qianqian Zhao, Yihui Xu, Shuyi Han, Huanjie Li, Yunshan Wang

**Affiliations:** ^1^ Institute of Medical Sciences, the Second Hospital of Shandong University, Jinan, Shandong, China; ^2^ Medical Integration and Practice Center, Cheeloo College of Medicine, Shandong University, Jinan, Shandong, China; ^3^ Research Center of Basic Medicine, Jinan Central Hospital, Shandong University, Jinan, Shandong, China

**Keywords:** SARS-CoV-2, COVID-19, neutralizing antibody, CD4+, CD8+, CD25+, CD69+, booster vaccine

## Abstract

**Introduction:**

Severe acute respiratory syndrome coronavirus 2 (SARS-CoV-2) is highly pathogenic to humans and has caused the ongoing coronavirus disease 2019 (COVID-19) pandemic. Vaccines are one of the efficient ways to prevent the viral infection. After COVID-19 vaccination, the monitoring of the dynamic change in neutralizing antibodies is necessary to determine booster requirements.

**Methods:**

We estimated the effectiveness of the inactivated vaccines by monitoring dynamic SARS-CoV-2 neutralizing antibodies for over 2 years. Additionally, we also investigated the activation of T lymphocytes (CD3+ T cells) after three doses of the inactivated vaccine.

**Result:**

The results showed that the rate of reduction of SARS-CoV-2 neutralizing antibody levels gradually showed after each booster dose. The IgG/IgM level at 9 months after the third vaccination were significantly higher than those at 6 months after the second dose (p<0.0001). The expression of CD25+T cell in 18–35 age group was significantly higher than that in the other groups. Nine months after the third dose (the time of last blood sample collection), the expression of CD25+T cell in the 18–35 age group was significantly higher than that at 6 months after the second dose. CD25+T cell in the 18-35 years old group was significantly higher than 6 months after the second vaccination.

**Conclusion:**

CD25, a late activation marker of lymphocytes and high-activity memory T cell subgroup, exhibited higher levels at the later stages after vaccination. COVID-19 booster vaccination in older adults and regular testing of SARS-CoV-2 neutralizing antibodies are recommended. Booster doses should be administered if the antibody level falls below the 30% inhibition rate.

## Introduction

The coronavirus disease 2019 (COVID-19) pandemic caused by severe acute respiratory syndrome coronavirus 2 (SARS-CoV-2) continues to cause significant morbidity and is a major burden on public health worldwide (1). According to cumulative reports, 89.63% of China’s total population has received the SARS-CoV-2 vaccines to date (2). SARS-CoV-2 infection has been effectively controlled because of vaccines development and initiatives to support mask wearing and social distancing (3). The following categories of COVID-19 vaccines have been approved for clinical trials: inactivated vaccine, live attenuated, vector, RNA, DNA, protein subunit, and virus-like particle (VLP). These vaccines have been effective and significantly mitigate COVID-19 symptoms and provide protection against serious and fatal infections (4). Inactivated vaccines have a good preventive effect on various mutations and are widely used in China (5). Three–doses of the inactivated vaccine have prevented high mortality in elderly people (> 60– year– old) due to Omicron infection (6). Based in an immunogenicity study, a superior protective efficacy of the inactivated vaccine is expected in the real-world settings (7). Therefore, it is crucial to investigate the level of sub-population immunity following inactivated vaccine administration.

After vaccination, the titers of binding and neutralizing antibodies decline over time (8). According to a preprint article, the neutralizing antibodies decreased significantly to 44.1% and 62.5% after the vaccination of the ChAdOx1 nCoV-19 (Oxford-AstraZeneca) and BNT162b2 (Pfizer-BioNtech) vaccines (9). A study with American veterans showed that the risk of infection increased significantly 6 months after vaccination (10, 11). To achieve the best protective efficiency of vaccine, individuals should perform long-term monitoring of the dynamic trends of SARS-CoV- 2-specific neutralizing antibodies and determine appropriate time points for booster vaccination.

In the first few months following vaccination, antibodies have been identified as the clear protective factor against infection (12). However, some studies showed that T and B cell responses also play an important role in protective immunity (13), even in the absence of a humoral immune response (14–16). B cells produce antibodies, and CD4+T cells have a series of auxiliary and effector functions. Although cells produce COVID-19 antibodies, CD4+T cells can differentiate into a series of helper cells and effector cell types, which can guide B cells, help CD8+ T cells, and recruit innate cells. Additionally, they have direct antiviral activity and can promote tissue repair (17). CD8+T cells kill infected cells. CD25 is a late activation marker of lymphocytes and have been reported to be related to the severity of COVID-19 infection (18). CD69 is a classical early marker of lymphocyte activation, and CD19 CD69 are closely related to heart failure caused by COVID-19 (5). T cell counts in critical patients, including CD3+T, CD4+T, CD8+T, CD25+CD4+, and CD19+B, were significantly lower in critical patients compared to those in the non-critical patients with COVID‐19 (19). However, the examination of CD25+T and CD69+T cells is still rare in vaccine-related research. In general, distinct T and B cell responses have been observed following vaccination in comparison to those in response to natural infection (20). Further studies on the immune cell response to inactivated vaccination are required.

Vaccinations have been successful in promoting humoral immunity. Nevertheless, antibody titers gradually decreased after vaccination, which leads to a decline in neutralizing activity (21). Therefore, periodic population follow-up on antibody quantification becomes increasingly relevant for immunological monitoring and COVID-19 pandemic management (22, 23). The gold standard for evaluating immunological responses to vaccination and infection is serological testing for SARS-CoV-2 antibodies (23).

In this study, we conducted questionnaires survey and long-term serum collection from volunteers to detect SARS-CoV-2 neutralizing antibodies and T-cell related immune subtypes. Dynamic SARS-CoV-2 S specific IgM, IgG, and neutralizing antibody changes after inactivated SARS-CoV-2 vaccinations as well as the neutralizing antibodies and their relevance to booster doses were assessed with the aim to optimize the dose timing, and COVID-19 vaccines’ protective efficiency.

## Materials and methods

### Study design and participants

The study was conducted in two parts. First, an online survey using the WeChat Questionnaire Star software were conducted. 424 healthy adults aged 18-70 years and healthy teenagers aged 12-18 years completed the survey. Second, we recruited 60 volunteers for the trial. They received three doses of the BBIBP-CorV (Sinopharm) or CoronaVac (Sinovac Biotech) COVID-19 inactivated vaccine. The boosting effect of the three vaccine doses was evaluated for safety, reactogenicity, and immunogenicity at 7, 14, 21, 28, and 56 days after vaccination. The study was conducted accordance with the guidelines of the Declaration of Helsinki and was approved by the Institutional Review Board of the Ethics Committee of Jinan Central Hospital (Ethical code No. D202111Ab). All participants provided written informed consents. Questionnaire and informed consent forms are available upon request.

### Design of public opinion survey

The questionnaire surveyed participants’ attitudes towards vaccines and enquired if they wanted to be vaccinated against COVID-19. This survey was divided into three parts: the basic characteristics of COVID-19 vaccine, vaccine awareness and vaccination history. The questionnaires were administered using WeChat Mini Program Questionnaire Star, and included participants’ sociodemographic characteristics, medical history, vaccination rates, and main reasons for vaccination. Additionally, questions on participants’ knowledge of immune responses after vaccination and the impact of the COVID-19 pandemic on vaccination. Participants selected their responses from a pre-determined set of options for each question. Information sheets were provided to explain the study.

### Specimen collection

Samples for trial were collected from 60 volunteers who agreed to be vaccinated against COVID-19. The volunteers were medical workers or hospital researchers, and provided nasopharyngeal and oropharyngeal swabs daily. Nasopharyngeal swabs and oropharyngeal swabs from volunteers were placed a sterile test tube containing 3 mL of virus preservation solution; all samples were tested for SARS-CoV-2 using PCR and confirmed as negative. During the study, none of participants experienced SARS-CoV-2 infection. In January 2021, blood samples were collected from volunteers without a history of COVID-19 vaccination, and then at every 7 or 15 days following the first, second and third dose of inactivated COVID-19 vaccine. Blood samples were collected until September 23, 2022. Owing to the frequency of blood sample collection, not all volunteers were able to provide the blood samples at each occasion of sample collection. At each time point, a minimum of 14 samples were collected.

### Laboratory analysis

SARS-CoV-2 RNA was extracted using the magnetic beads method, according to the instructions of the nucleic acid extraction kit followed by testing with RT-PCR following the steps of the kit in a tertiary protection laboratory (Shanghai Zhijiang Biotechnology Co., Ltd, Shanghai, China). At each sample collection, two tubes of blood were collected from each volunteer. Procoagulant tube of blood sample was centrifuged at 3,000×g for 10 min at 25 °C to isolate serum for antibody testing, the other blood sample was anticoagulated and sorted using flow cytometry. We used the SARS-CoV-2 neutralizing antibody test kit (Shandong LaiBo Biotechnology Co., Ltd, Shandong, China) competitive enzyme linked immunosorbent assay (ELISA) principle to qualitatively detect SARS-CoV-2 neutralizing antibody in human serum. First, biotin-labeled ACE2 working solution was added to the wells of the streptavidin-coated plate. After a warm bath, the unbound biotin-labeled ACE2 was removed by washing. The serum, positive control, and negative control were added to the plate wells followed by the addition of binding enzyme. After warm bath, the enzyme was washed, and a chromogenic solution was added. After the reaction termination, absorbance at 450 nm/630 nm dual wavelengths, was detected using an enzyme marker. The absorbance of the sample was negatively correlated with the titer of the neutralizing antibody. Baseline and serum levels of SARS-CoV-2 S-specific binding antibodies were determined using a WanTai antibody detection kit (WANTAISARS-CoV-2NAbsELISA, Beijing, China). The kit uses recombinant samples and the novel coronavirus coated antigen to detect chemical immunofluorescence signals. The relative light results positively correlated with the level of SARS-COV-2 S-specific antibodies in the serum. Intracellular cytokine staining was used to measure to quatify the T cell response. The fluorescence-labeled antibodies to CD4+, CD8+, CD69+, and CD25+ in the reagent bind to the antigens in the cells to be tested to prepare single cell or suspension samples. For the assay, 10 μL of the reagent was added into the flow tube, followed by 100 μL of sample. This mixture was thoroughly mixed using a vortex oscillator and incubated for 15 min at 25°C in the dark. Then, 2 mL of hemolysin was added after incubation, thoroughly mixed. The mixture was then left for complete fragmentation of red blood cells and centrifuged for 5 min at 1500 ×g at 25°C. The supernatant was discarded, and 300 μL of PBS was added to resuspend the sample before the machine assay for absolute counting. Data were acquired using flow cytometry (LSR II with FACS Diva version 8.0; BD Biosciences, Franklin Lakes, NJ, USA) and analyzed using FlowJo version 10 software (BD Biosciences, Ashland, USA).

### Statistical analysis

For descriptive statistics, frequencies and percentages were calculated for each question of the survey. SARS-CoV-2 neutralizing antibodies were calculated from the inhibition rate according to the absorbance value detected by using the enzyme marker. OD values of negative control and sample were used to calculate the inhibition rate of the testing sample, whereas OD values of the positive control were used to evaluate the validity of the test. The equation to calculate neutralizing antibody inhibition rate in the testing samples is as follows:


$$\text{Neutralizing antibody inhibition rate}=\left(1-\frac{\text{OD value of sample}}{\text{Mean value of OD in negative control}}\right)\times 100\%$$

When the inhibition rate of the sample is <30%, the neutralizing antibodies are considered negative.

Neutralization and antibody inhibition rates at each time point were statistically analyzed, and the mean value and 95% confidence interval were obtained. The sample mean, the significance level, and the sample standard deviation is χ, α, and S, respectively. which are estimates of the normal population mean μ and standard deviation σ. The linear interval method was used to quantify the uncertainty, with 95% as the quantization standard, i.e., 2σ. The mean neutralizing antibody inhibition rate and 95% CI difference interval of the samples at different time points were calculated using following formula:


$$\bar{\chi }-\frac{\sigma }{\sqrt{n}}z_{\frac{a}{2}}\leq \text{μ}\leq \bar{\chi }+\frac{\sigma }{\sqrt{n}}z_{\frac{a}{2}}$$

Statistical analyses were performed using Prism 7 software (GraphPad). Appropriate statistical tests were used for each comparison after determining the normality of the data using the Shapiro–Wilk and Kolmogorov–Smirnov tests at a significance level of α = 0.05. Comparisons of flow cytometry cell frequencies for mice studies were measured using the two-way analysis of variance test with the Holm-Sidak multiple–comparison test or an unpaired t-test with * *p* < 0.05, ** *p* < 0.01, *** *p* < 0.001, and **** *p*<0.0001 denoting statistical significance.

## Results

### Experimental process

**Figure 1:**
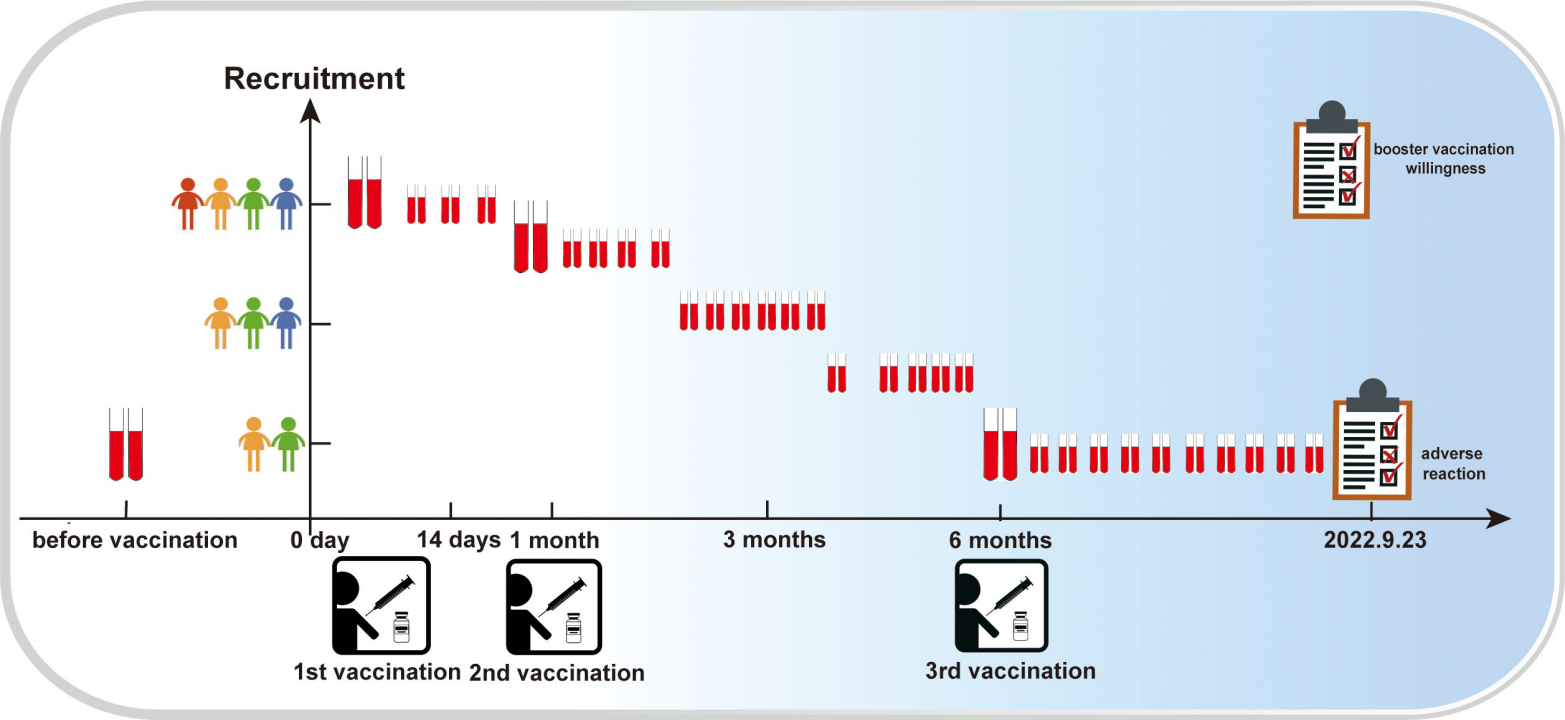
Research process flow diagram. The vertical coordinate represents the number of participants; the higher the number of dolls, the higher the number of participants. The horizontal coordinate represents time; the red blood collection tube indicates blood collection at the given coordinate point.

To investigate the adverse reactions public after vaccination and participant attitudes towards COVID-19 booster shots, we conducted two public opinion surveys during the 2 years of the pandemic. To study the immunogenicity and reactogenicity of inactivated SARS-CoV-2 vaccines, we collected blood samples from volunteers before they received the vaccine starting in January 2021, and every 7 or 15 days after receiving of the inactivated COVID-19 vaccine until September 23, 2022. Neutralizing antibodies, total antibodies (IgG+ IgM), and CD4+, CD8+ and other antigens responses in T cells and B cells were evaluated. The study flow diagram is shown in **Figure 1**.

### Respondent characteristics

To study the relationship among vaccine side effects, number of doses, and neutralizing antibodies, we administered a questionnaire on adverse reactions. The characteristics of the 424 participants including sex, age, health, illness, and vaccination conditions were analyzed. As shown in **Table 1**, six of the 424 participants have not received any vaccine, seven received only one dose, 86 received two doses, and 325 received three doses. The number of participants willing to accept a fourth booster dose was 337 (80.62%). The results showed that 385 participants were healthy (90.8%), and the remaining participants had various conditions, including hypertension (28 participants), diabetes (15 participants), and coronary heart disease (4 participants). Twelve participants were diagnosed with chronic respiratory disease, three with immunodeficiency disease, and 16 with other illnesses.

**Table 1:** Characteristics of questionnaire participants.

Characteristic	Group	n	%
Gender	Male	182	42.92%
	Female	242	57.08%
Age	12-18	2	0.47%
	19-25	108	25.47%
	26-40	207	48.82%
	41-60	100	23.58%
	over 60 years old	7	1.65%
Healthy	Good	385	90.80%
	General	36	8.49%
	Poor	3	0.71%
Basic illness	Hypertension	28	6.60%
	Diabetes	15	3.54%
	Coronary heart disease	4	0.94%
	Chronic respiratory disease	12	2.83%
	Immunodeficiency disease	3	0.71%
	Obesity	47	11.08%
	Other illnesses	16	3.77%
	None of the above	328	77.36%
Vaccination doses	1 dose	7	1.65%
	2 doses	86	20.28%
	3 doses	325	76.65%
	Not vaccinated	6	1.42%
Adverse reactions to other vaccines		99	23.68%
Willing to continue booster vaccinations		337	80.62%
Still suffering from COVID-19 after being vaccinated		5	1.20%

n, number of participants; % = percentage of the total number of participants in this group.

### Adverse reactions after COVID-19 vaccination

**Figure 2:**
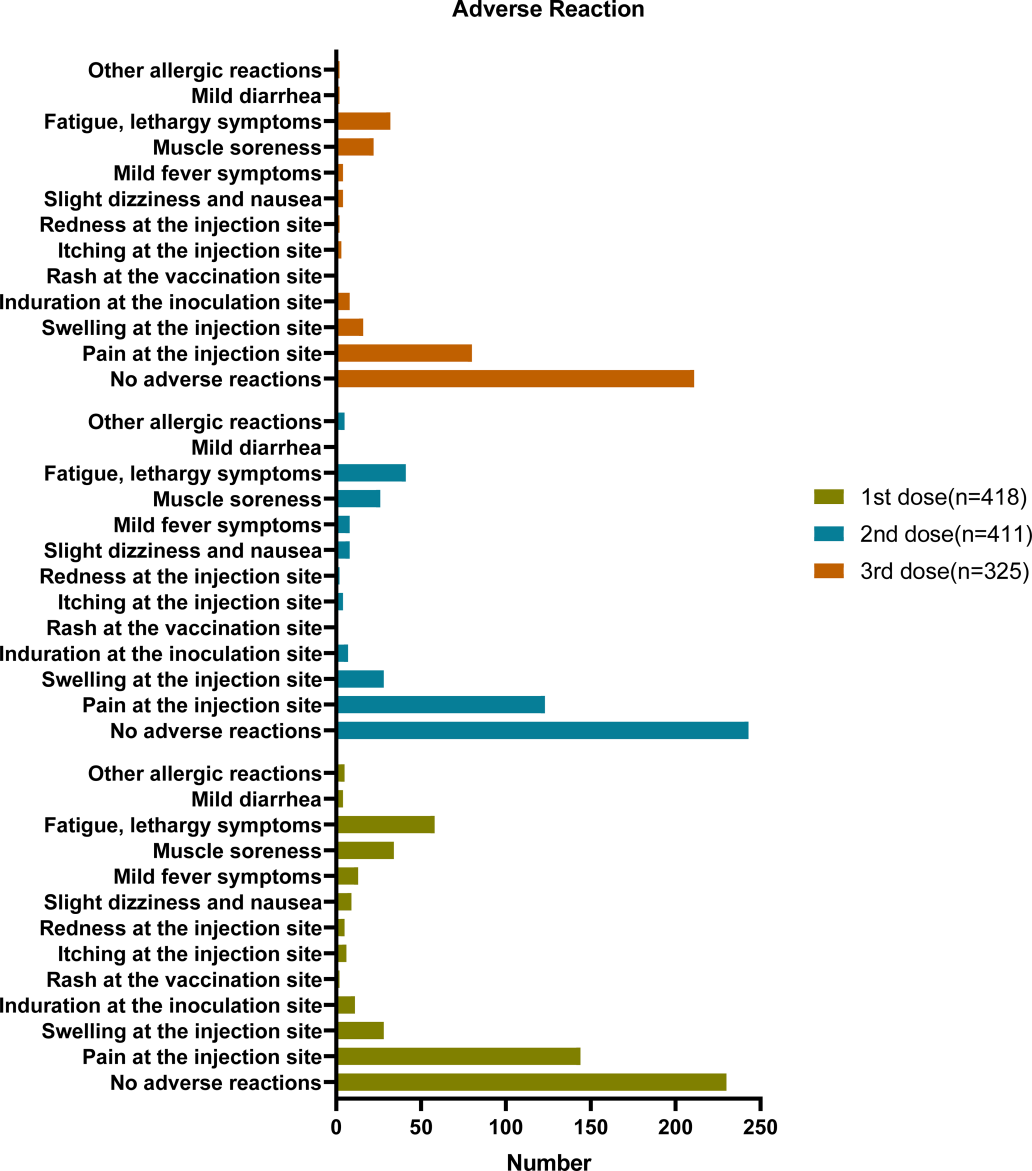
Adverse reaction based on questionnaire responses. six of the 424 participants have not received any vaccine (418 + 6 = 424), seven received only one dose (411 + 6 + 7 = 424), 86 only received two doses, and 325 received three doses (325 + 86 + 6+7 = 424).

The questionnaire survey investigated the adverse reactions following vaccination; most participants reported no adverse reactions (**Figure 2**; **Table 2**). The proportion of patients with no adverse reactions increased as the number of vaccinations increased. Overall, 55.02%, 59.71%, and 66.14% of participants had no adverse reactions after the first, second, and third dose, respectively. **Table 2**; **Figure 2** showed the adverse reactions of the participants, including injection site pain, injection site swelling, fatigue, lethargy, and muscle soreness. After three injections, the main adverse reaction was pain at the injection site (34.35%, 30.22%, and 25.08% after the first, second, and third dose, respectively). The second most common adverse reactions were fatigue and drowsiness, accounting for 13.88%, 10.07%, and 10.03% of adverse reactions after the first, second, and third dose, respectively. The third most common adverse reaction was muscle soreness (8.13%, 6.39%, and 6.9% after the first, second, and third dose, respectively). Other adverse reactions were fewer, with mild dizziness and nausea accounting for only 2.15%, 1.97%, and 1.25% after the first, second, and third rounds of inoculation, respectively. After three rounds, 3.11%, 1.97% and 1.25% participants had fever symptoms, respectively. 6.7%, 6.88%, and 5.02% of the participants had an injection site swelling after the first, second, and third injections, respectively. Other adverse reactions are shown in **Table 2**; **Figure 2**. After the implementation of the pandemic prevention and control measures, the follow-up survey showed that 95% of the 424 participants have been infected by SARS-CoV-2 by January 2023, and the symptoms of infection showed no statistical difference by vaccination status or side effects (date not shown).

**Table 2:** Adverse reaction of questionnaire participants.

Adverse Reactions	1st dose		2nd dose		3rd dose	
	n	%	n	%	n	%
No adverse reactions	230	55.02%	243	59.71%	211	66.14%
Pain at the injection site	144	34.45%	123	30.22%	80	25.08%
Swelling at the injection site	28	6.70%	28	6.88%	16	5.02%
Induration at the inoculation site	11	2.63%	7	1.72%	8	2.51%
Rash at the vaccination site	2	0.48%	0	0%	0	0%
Itching at the injection site	6	1.44%	4	0.98%	3	0.94%
Redness at the injection site	5	1.20%	2	0.49%	2	0.63%
Slight dizziness and nausea	9	2.15%	8	1.97%	4	1.25%
Mild fever symptoms	13	3.11%	8	1.97%	4	1.25%
Muscle soreness	34	8.13%	26	6.39%	22	6.90%
Fatigue, lethargy symptoms	58	13.88%	41	10.07%	32	10.03%
Mild diarrhea	4	0.96%	1	0.25%	2	0.63%
Other allergic reactions	5	1.20%	5	1.23%	2	0.63%

n, number of participants; % = percentage of the total number of participants in this group.

### Dynamic serosurvey of SARS-CoV-2 neutralizing antibodies concentration

**Figure 3:**
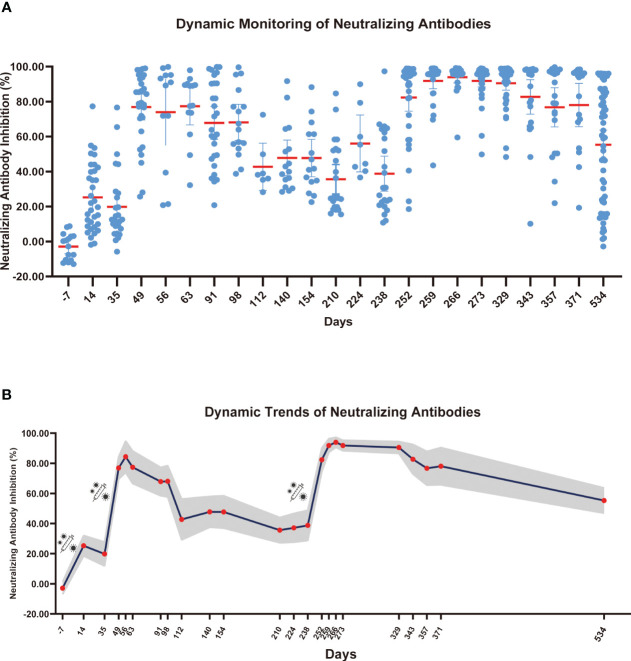
Dynamic monitoring of SARS-CoV-2 neutralizing antibodies. **(A)** Each dot represents an individual’s neutralizing antibody inhibition rate, and the red horizontal line represents the average level of each group. **(B)** The gray part represents the 95% confidence interval of the mean neutralizing antibody level in this time, and the red dot represents the average number of neutralizing antibodies for this period.

To comprehensively access the effect of COVID-19 vaccine, we analyzed the dynamic trend of COVID-19 neutralizing antibodies over time. Starting from January 2021, blood samples were collected from volunteers with no prior history of COVID-19 vaccination. Blood was collected at every 7 or 15 days or more after the volunteers were vaccinated. Blood samples were collected till September 23, 2022. The research continued for 2 years from the first vaccination dose and the participants were all healthy people. Collection number of blood samples once a week or at two weeks is difficult, especially over 2 years period. The range of participants remained the same, but a few people were guaranteed to contribute blood samples every time point. The sample size we collected at each time point was not fixed, with a minimum of 14 participants and maximum of 60 participants per group. The detailed sample collection times are listed in **Table S2**; **Figure 3**. The overall trend showed meaningful differences despite the limited number of samples at each collection.

As shown in **Figure 3**, neutralizing antibodies were gradually produced 14 days after the first COVID-19 vaccine dose, and the inhibition rate of neutralizing antibodies reached 25.27%. However, after 35 days, the inhibition rate of neutralizing antibodies decreased to 19.83%. The inhibition rate of neutralizing antibodies reached a second peak of 84.33% at 21 days after the second dose (56 days after the first dose), and the inhibition rate slowly decreased over 120 days after the second dose. At 175 days after the second dose, the neutralizing antibody inhibition rate decreased to 35.60%. After the third booster vaccine dose was administered, the inhibition rate of neutralizing antibody reached 93.96% at 21 days and remained above 90% for 90 days after inoculation. On September 23, 2022, we collected blood samples from volunteers for the last time and evaluated the neutralizing antibody inhibition rate. The neutralizing antibody level decreased to 55.26%. We found that the rate of reduction in SARS-CoV-2 neutralizing antibody levels gradually declined after each booster dose. Given that neutralizing antibodies declined over time, we speculate that people may require an additional COVID-19 booster shot to maintain antibody levels and achieve the best protective effect of the vaccine.

After the last blood collection, we detected the level of neutralizing antibodies and SARS-CoV-2 S-specific binding antibodies (IgG + IgM) in serum analyzed those samples by age group. As shown in **Figure S1A**, the SARS-CoV-2 S-specific IgG + IgM levels in volunteers aged 55–70 years had dropped below the critical value., In the 18–35 years age group, the antibody levels were significantly higher than those in the 35–55 years age group (*p*<0.05), and significantly higher than those in the 55–70 age group (*p*<0.0001). As shown in **Figure S1B**, the levels of neutralizing antibodies in 18-35 years age group were significantly higher than those in the 55–70 years age group (*p*<0.0001). SARS-CoV-2 antibody levels decreased with age and were maintained longer in young people. In our previous study (24), the expression of IgG + IgM in volunteers aged 18–35 years was detected on day 7 before the third dose after 1 month of vaccination. The IgG + IgM level at 9 months after the third dose was significantly higher than those at 6 months after the second dose (*p*<0.0001). Additionally, the result showed that the IgG + IgM level could be maintained for longer after booster vaccination in most people in this group compared to those in the older age group.

### Individual dynamic surveillance and heterogeneity analysis of COVID-19 vaccines

**Figure 4:**
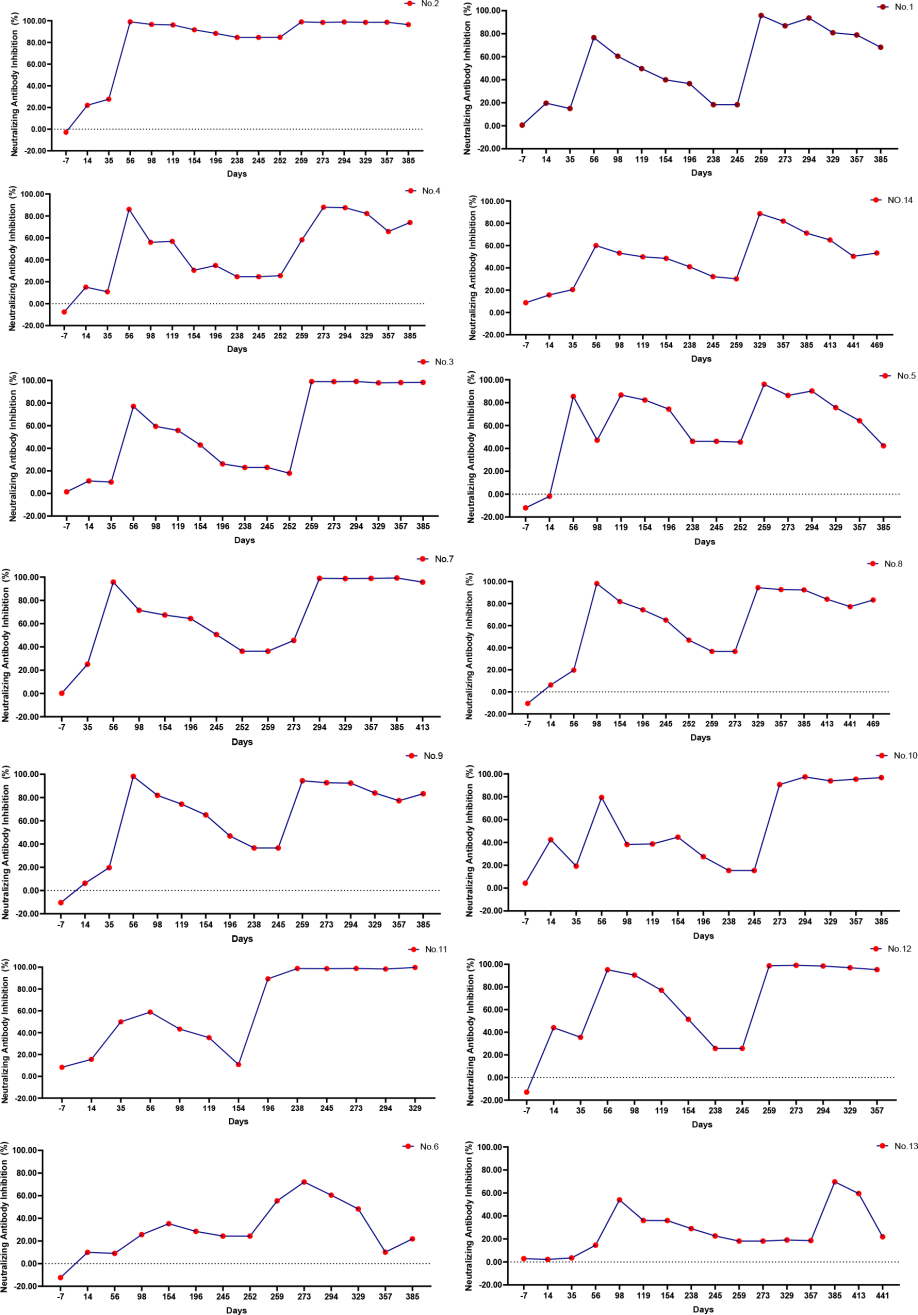
Dynamic monitoring of individual neutralizing antibodies. During the study, only 14 volunteers participated in consecutive blood draws. The legend in the upper right corner represents the number of participants. According to the continuous dynamic trend of SARS-CoV-2 neutralizing antibody antibodies in the 14 participants, the antibody change trends could be roughly divided into approximately 4 categories. [2], [3,5,7,8,9,10,11,12], [1,4,14], [6,13].

To study the heterogeneity of the COVID-19 vaccine response in different individuals, we recruited volunteers who had not yet received the first COVID-19 vaccine dose; blood samples were collected from 14 individuals at regular (10 men and 4 women) at intervals from before the first dose to 6 months after the third dose. We conducted a separate survey of vaccination information for these 14 volunteers, and the results are shown in **Table S1**. Five of the ten men were aged 26–40 years, and the remaining five were aged 40–55 years. Of the 4 women, two were aged 26–40 years and 2 were aged 40–55 years. One of these men aged 40–55 years had obesity. We recorded the volunteers’ adverse reactions after vaccination. Most of them had showed no adverse reactions; only a few experienced pain at the vaccination site. Adverse reactions in three volunteers were alleviated with the subsequent vaccine doses, whereas adverse reactions in another three volunteers increased with the subsequent doses (**Tables S1**, **S2**). We further analyzed the continuous dynamic trend of SARS-CoV-2 neutralizing antibodies in the 14 participants. As shown in **Figure 4**, the antibody change trends in volunteers could be divided into approximately 4 categories. The antibody levels of volunteer 2 remained stable after the second vaccine dose. The trend in neutralizing antibodies was the same in volunteers 3, 5, 7, 8, 9, 10, 11 and 12. The inhibition rate slightly increased after the first vaccine dose, weakened, and then increased again until antibody levels stabilized after the third vaccine dose. The levels of neutralizing antibodies in volunteers 1, 4, and 14 increased after vaccination. The peak pattern showed a continuous fluctuation, and finally decreased slowly after the third vaccine dose. In volunteers 6 and 13, the levels of neutralizing antibodies increased only modestly after vaccination, and then decreased quickly.

### Response of CD4+T and CD8+ T cells at different age groups

**Figure 5:**
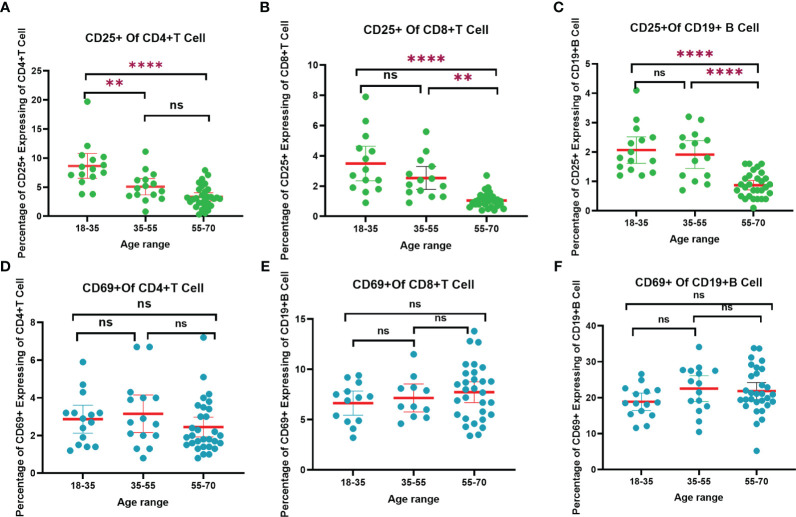
The trend of CD4+, CD8+ T cells and CD19+ B cells responses to SARS-CoV-2 vaccination in different age range after 9 months of the third dose. **(A)** CD25+ of CD4+ T cells trend chart. **(B)** CD25+ of CD8+ T cells trend chart. **(C)** CD25+ of CD19+ B cells trend chart. **(D)** CD69+ of CD4+ T cells trend chart. **(F)** CD69+ of CD8+ T cells trend chart. **(E)** CD69+ of CD19+ B cells trend chart. “**”*p* < 0.01; “****”*p* < 0.001. “ns”, not significant.

Understanding the complex mechanisms of SARS-CoV-2 immunological memory is crucial to understanding the protective immunity against SARS-CoV-2 reinfection and secondary COVID-19 persistence (25). In the current study, we divided the volunteers into three age groups and analyzed their CD4+ and CD8+ levels after vaccination. At the final blood collection, the 35–55 years age group had the highest CD4+ level and the lowest level of CD8+ levels, whereas these levels in the other two age groups were the same. Additionally, we measured the CD25+ and CD69+ expression. As shown in **Figures 5A–C**; **Table S3**, the expression of CD25+ was the lowest of volunteers aged 55–70 years and the highest in volunteers aged 18–35 years. The expression of CD25+CD4+ T cells in the 18-35 years age group was significantly higher than that in the 35–55 years age group (*p*<0.01), and higher than that in the 55–70 years age group (*p*<0.001).The expression of CD25+CD8+ T cells in 55-70 years age group was significantly lower than that in the 35-55 years age group (*p*<0.01), and significantly lower than that in the 18–35 (*p*<0.0001). The expression of CD25+CD19+ B cells in 55-70 age group was significantly lower than 35-55 (*p*<0.001), and extremely significant lower than 18-35 (*p*<0.0001) (**Figure 5C**). Conversely, the expression of CD69+CD19+ B cells in 35-55 age group was higher than the other two groups (**Figure 5F**). These results suggest that participants in the younger age group were able to better suppress cellular inflammatory cytokine storms for a longer period than did those in older age groups after vaccination. In **Figure 5**, T cells and B cells of those in the younger age group remained more activated than those in older age groups. This finding might be associated with less efficient cellular immune responses in older adults as previously reported (26). In the preliminary analysis of our study, the dynamic changes in CD25+ and CD69+ were detected before and after the third booster (24). After 9 months of the third dose (the last blood sample collection), the expression of CD25+ CD4+ T cells in the 18–35 years age group was significantly higher than that at 6 months after the second dose (*p* < 0.001), and was significantly higher than a months after the third dose (*p* < 0.01). The results for CD25+ CD8+ T cells were consistent with those for CD25+ CD4+ T cells. The reason might be that CD25 is a late activation marker of lymphocytes and a highly active memory T cells subset. CD25 has been shown to be at a high level 9 months after a booster dose (27). CD69 is a hallmark of early activation of T cells (28). As shown in **Figures 5D–F**, the patterns of CD69+ levels in different age groups were opposite to those of CD25+, indicating that the immunosuppressive ability of the younger participants was lower than that of the participants in the older age groups. At 9 months after the third dose, the expression of CD69+CD8+T cells in the 18–35 years age group was significantly higher than that at 6 months after the second dose *(p* < 0.01), and significantly higher than that at 1 months after the third dose (*p* < 0.01). (All the details of the corresponding figures are in **Table S3**).

## Discussion

Globally, 764,474,387 confirmed cases of COVID-19, including 6,915,286 deaths, have been reported to WHO as of 26 April 2023. As of April 2023, a total of 13,325,228,015 vaccine doses have been administered (WHO Coronavirus (COVID-19) Dashboard). Three years have passed since the COVID-19 pandemic outbreak, and SARS-CoV-2 has persisted in humans and continues to mutate and spread (29).Inactivated vaccines can trigger various immune reactions, and the development and use of effective vaccines requires a thorough understanding of the immunological reactions. However, the immune system profiles after vaccination are still poorly understood (30). The present study used a questionnaire survey to explore the willingness to receive COVID-19 booster doses and the reasons for hesitation that influence people’s vaccination decisions. Additionally, to identify an optimal time for booser doses, we investigated the relationship of vaccine administration and immune system changes, including SARS-CoV-2 neutralizing antibodies, T cells and B cells. It is critical to investigate the effect of risk perception on individual immunity levels and preventive behavioral responses. In this study, we collected volunteer serum samples over time and administered questionnaires to study the correlation between SARS-CoV-2 neutralizing antibodies and T cell-related immunological hypo-type variables. The detection of neutralizing antibodies and their relationship to booster dose timing, as well as monitoring dynamic SARS-CoV-2 specific IgM, IgG, and surrogate neutralizing antibody levels in response to inactivated vaccines were analyzed to optimize vaccination, timing of booster doses, and the protective effectiveness of the vaccines.

In December 2021, we (31) conducted the first public opinion survey on booster vaccinations, which showed that 366 of 395 participants in the study cohort (92.66%) were willing to receive a third dose of the COVID-19 vaccine. In addition, 84% of participants expressed trust in the COVID-19 vaccine. We conducted a second survey in June 2022 with additional 424 volunteers. We observed significant decrease in the willingness to receive the fourth dose. Despite the mild side effects of COVID-19 vaccination, these contributed to hesitancy in older people. Global vaccination acceptability and hesitation are assessed using various measures and indices (31, 32). We listed six main reasons for non-vaccination in the questionnaire, namely, concerns about the vaccine’s efficacy, safety, and/or side effects; belief that the participant does not need it; lack of trust in the vaccines owing to their quick development; distrust in the pharmaceutical industry; and belief that COVID-19 is not a serious threat. Public health authorities and experts have not effectively addressed the underlying issues of vaccine reluctance (33). In view of this, it is very important to study immunology in reponse to the vaccination.

We studied the continuous dynamic changes in neutralizing antibodies in response to the COVID-19 vaccine. In general, the neutralizing antibodies increased and then declined, especially in older people. Similar results have been reported in previous studies (34–37) Additionally, less than 10% protection against symptomatic disease due to the Omicron variant was reported in the UK at 25 weeks following a two dose vaccine regimen (38). Here, we found a significantly decreased neutralizing activity against the Omicron variant in the convalescent and two-dose BBIBP-CorV vaccination group, which has also been confirmed by others and similar to our preliminary real-world data (39–41). From the perspective of individual differences, the participants could be divided into four categories according based on changes in the neutralizing antibodies after vaccination. In the first category, with the increased number of boosters, the neutralizing antibodies gradually increased. In the second category, only temporary neutralizing antibodies were produced after vaccination, which decreased rapidly by the next does. In the third category, the neutralizing antibodies were never increased after vaccination. The fourth category included the people who were able to maintain a peak since vaccination. Therefore, we could choose the best time point of vaccination according to individual differences in response to the vaccine.

Understanding the immunological memory of vaccine effects may necessitate an investigation of its diverse components. CD8+ T, and CD4+ T cells are different cell types with respective immune memory kinetics. The vaccine immunological response thus varied among different people. Additionally, there are special requirements for different populations and health conditions. In this study, we observed that the neutralizing antibody levels were maintained for various duration—extremely long, continuously high; however for particular groups, such as olderly people, it was very challenging to raise the antibody levels. Additionally, we examined the T-cell immunological components in effort to determine the reason. Higher age was related with a greater decline of CD25+ in T cells. An analysis of participants who received only one dose of ChAdOx1 showed that 14 days after immunization, T cells from CMV donors had a higher terminally differentiated profile of CD4+ T cells and CD8+ T cells, fewer IL-2R (CD25), and polyfunctional CD4+ T cells (42). In addition to triggering antibody responses, SARS-CoV-2 leaves long-lasting positive (i.e., stimulation of T cells) and potentially negative (i.e., decrease of neutrophils) imprints in the cellular immune system (43). This explains fewer CD25+ T cells in older people. A report showed that CD4+CD25+ cells triggered other cells to secrete immunosuppressive cytokines in an indirect manner (44). CD25+ B cells are immune response enhancers and strictly control the regulation of CD25 expression (45). In addition, CD25+ B cells are covered with immunoglobulins for the interaction with antigen that triggers subsequent T-cell activation (46). CD25+ cells are a highly active subset of memory T cells that may play a role in controlling inflammation via anti-inflammatory Th2-type deviation (27). This may be one of the reasons for the increase of CD25+ level in the younger age group with further doses. CD69+ is a sign of T cell activation (47). It can regulate the adaptive immune response through different mechanisms, including the prevention of Th17 differentiation and down regulation of proinflammatory cytokines (5). CD69+ is required for the trafficking of effector CD4+T cells to the bone marrow, particularly for their relocation and the persistence of their interaction with stromal cells as memory T helper cells (47). It is known that virus infection results in polyclonal activation of B cells, whose activation marker was the upregulation of the CD69+ B cell. The process is dependent on CD4 T cells (48, 49). As in our results, CD69+ in the young age group significantly increased with further doses and time since vaccination.

This study has some limitations. Although the pseudo-virus neutralization assay is the method of choice for measuring the neutralizing antibody levels, it requires considerable time and effort and has poor throughput, making it unsuitable for regular clinical testing. Additionally, we investigated whether a surrogate of neutralizing antibodies levels calculated using approved chemiluminescence assays correlated with the neutralizing antibody titers obtained from a pseudo-virus neutralization experiment. Meeting the demands for regular blood collection was challenging for participants and their compliance was limited. The small number of samples is one of the limitations of the present study.

## Conclusion

Based on the dynamic analysis of neutralizing antibodies of the novel coronavirus, immunological analysis and questionnaire survey questionnaire responses, the following conclusions and suggestions are drawn. CD25, a late activation marker of lymphocytes and a high-activity memory T cell subgroup, increases with time after COVID-19 booster vaccination in the older adults and regular testing of SARS-CoV-2 neutralizing antibodies are recommended. The booster doses should be administered if antibody levels fall below the 30% inhibition rate. Moreover, it is necessary to develop novel targeted vaccines against mutated strains.

## Data availability statement

The original contributions presented in the study are included in the article/**Supplementary Material**. Further inquiries can be directed to the corresponding authors.

## Ethics statement

The studies involving human participants were reviewed and approved by Institutional Review Board of the Ethics Committee of Jinan Central Hospital. The patients/participants provided their written informed consent to participate in this study.

## Author contributions

HL and YWu acquired and analyzed the data. HL and YWa supervised the research. PH, YX and YWu designed the study and wrote the manuscript. MX, QZ and SH were responsible for data curation. All authors contributed to the article and approved the submitted version.
