# Supplementary material for: Immunogenicity and reactogenicity of inactivated SARS-CoV-2 vaccines in healthy adults
Source: Front Immunol. 2023 Jul 25;14:1152899. doi: 10.3389/fimmu.2023.1152899 (PMC10407550; doi:10.3389/fimmu.2023.1152899)
Supplement: Supplementary file 1 [file DataSheet_1.docx]

**Immunogenicity and Reactogenicity of SARS-CoV-2 CoronaVac**

**Vaccines in Healthy Adults**

**Supplementary Material**


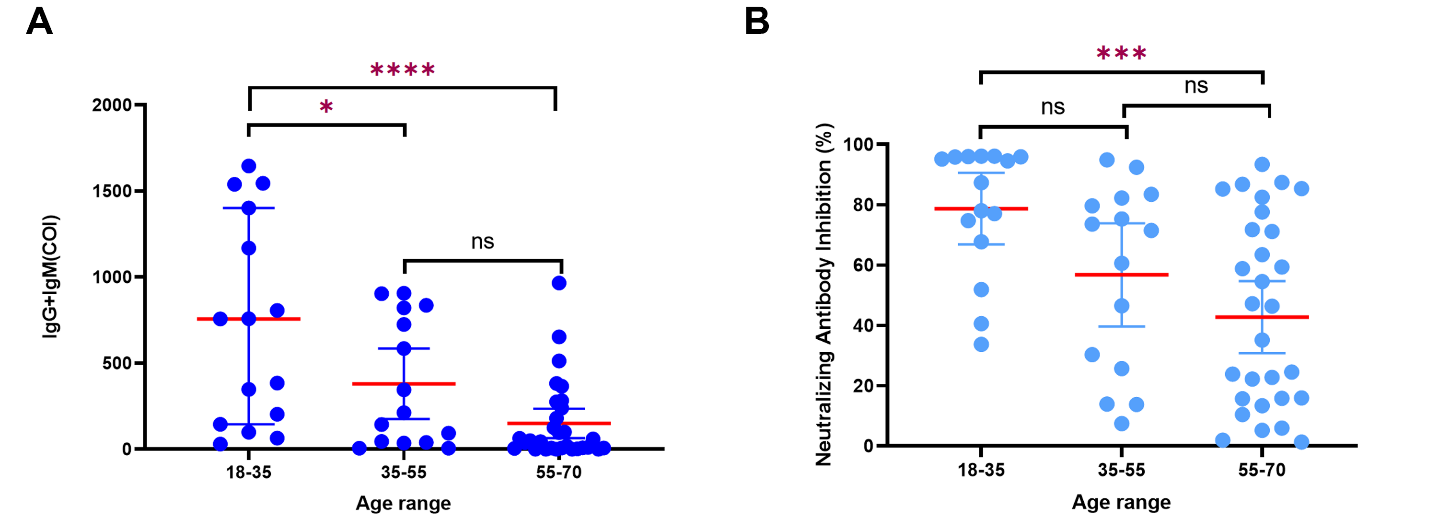
 **Figure S1.** SARS-CoV-2 S-specific binding antibody and SARS-CoV-2 neutralizing antibody in different age range

Note："**" *P* ＜0.01; "***" *P* ＜0.001; "****" *P* ＜ 0.001. "ns" not significant.

*
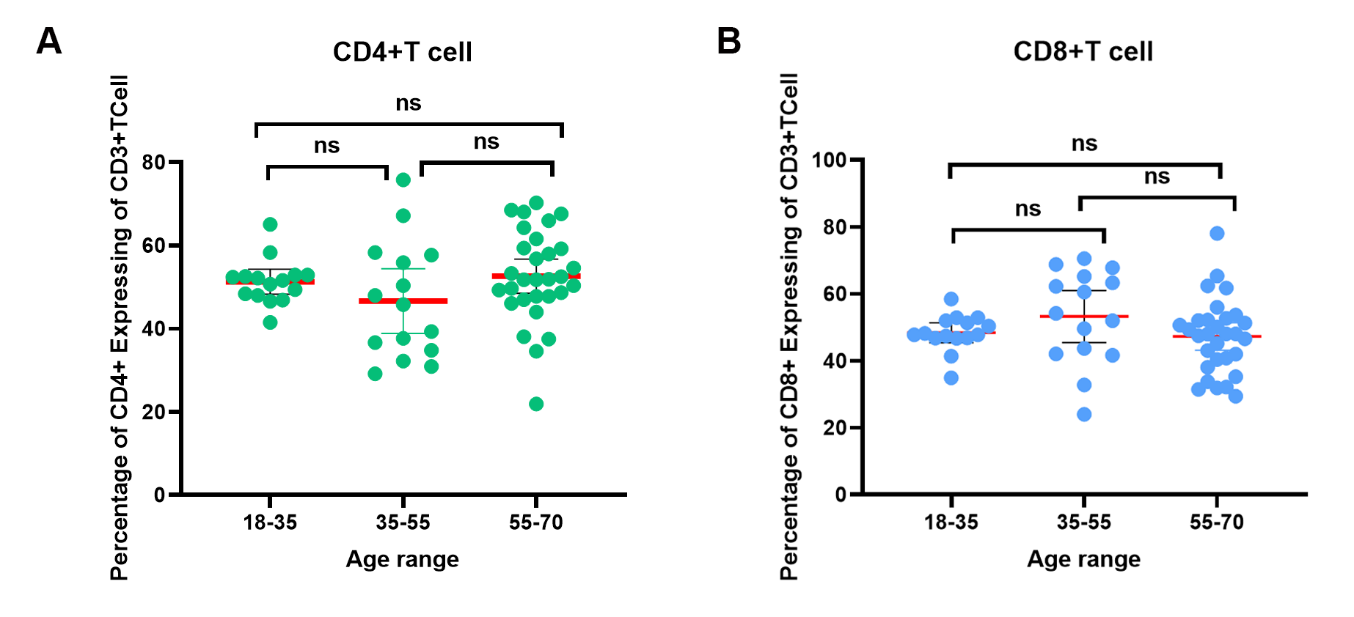
*

**Figure S2.** CD4+and CD8+ in different age range

Note："**" *P* ＜0.01; "***" *P* ＜0.001; "****" *P* ＜ 0.001. "ns" not significant.

**Table S1.** Characteristics and adverse reactions of clinical trials volunteer

| **Gender** | **Age** | **Basic illness** | | **Adverse reactions after 1st dose** | **Adverse reactions after 2nd dose** | **Adverse reactions after 3rd dose** | **The trend of adverse reactions** |
| --- | --- | --- | --- | --- | --- | --- | --- |
| **Male** | 26-40 | | Healthy | Asymptomatic | Asymptomatic | Asymptomatic | Asymptomatic |
|  |  |  | Healthy | Muscle ache | Muscle ache | Fatigue and lethargy | Same level |
|  |  |  | Healthy | Fatigue and lethargy | Asymptomatic | Asymptomatic | Decrease |
|  |  |  | Healthy | Asymptomatic | Asymptomatic | Asymptomatic | Asymptomatic |
|  |  |  | Healthy | Asymptomatic | Pain at the injection site | Pain at the injection site | Increase |
|  | 40-55 | | Healthy | Pain at the injection site | Pain at the injection site | Pain at the injection site | Increase |
|  |  |  | Healthy | Fatigue and lethargy | Asymptomatic | Asymptomatic | Decrease |
|  |  |  | Healthy | Swelling at the injection site | Swelling at the injection site | Swelling at the injection site | Increase |
|  |  |  | Healthy | Asymptomatic | Asymptomatic | Asymptomatic | Asymptomatic |
|  |  |  | Diabetes | Asymptomatic | Asymptomatic | Asymptomatic | Asymptomatic |
| **Female** | 26-40 | | Healthy | Asymptomatic | Asymptomatic | Asymptomatic | Asymptomatic |
|  |  |  | Healthy | Pain at the injection site/Fatigue and lethargy | Asymptomatic | Asymptomatic | Decrease |
|  | 40-55 | | Healthy | Pain at the injection site | Pain at the injection site | Pain at the injection site | Decrease |
|  |  |  | Healthy | Asymptomatic | Asymptomatic | Asymptomatic | Asymptomatic |

**Table S2.** Dynamic changes of population neutralizing antibody mean.

| ***days after vaccination (*)*** | ***means*** | ***95%CI*** | ***The number of participants(n)*** |
| --- | --- | --- | --- |
| -7 | -2.93813 | 1.09,-6.96 | 16 |
| 1st dose | | |  |
| 14 | 25.27588 | 32.06,18.49 | 34 |
| 35 | 19.83741 | 27.79,11.89 | 27 |
| 2nd dose (1st dose) | | |  |
| 14 (49) | 76.90452 | 84.3,69.51 | 32 |
| 21 (56) | 84.3325 | 94.78,73.88 | 14 |
| 28 (63) | 77.38071 | 88.13,66.64 | 14 |
| 56 (91) | 67.835 | 77.24,58.43 | 29 |
| 63 (98) | 68.08063 | 78.43,57.73 | 17 |
| 77 (112) | 42.7 | 56.23,29.17 | 14 |
| 105 (140) | 47.76688 | 57.98,37.56 | 16 |
| 119 (154) | 47.696 | 58.38,37.01 | 24 |
| 175 (210) | 35.6075 | 43.98,27.23 | 24 |
| 189 (224) | 37.11091 | 46.65,27.57 | 24 |
| 203 (238) | 38.79348 | 48.81,28.77 | 23 |
| 3rd dose (1st dose) | | |  |
| 7 (252) | 82.33794 | 90.15,74.52 | 34 |
| 14 (259) | 91.828 | 96.31,87.35 | 30 |
| 21 (266) | 93.9636 | 97.26,90.67 | 25 |
| 28 (273) | 91.84649 | 95.31,88.39 | 37 |
| 84 (329) | 90.5141 | 94.47,86.56 | 39 |
| 98 (343) | 82.69136 | 92.54,72.84 | 22 |
| 112 (357) | 76.7225 | 87.9,65.54 | 20 |
| 126 (371) | 78.04412 | 90.44,65.65 | 21 |
| 289 (534) | 55.2635 | 63.66,46.86a | 60 |

(*)*The number in parentheses represents the time interval since the first dose.

**Table S3. The Description of the Statistical Test**

Note：Data are shown as the mean and standard deviation (SD) of variables. Tukey's multiple comparisons test, compare the mean of each column with the mean of every each other column."**" *P* ＜0.01; "***" *P* ＜0.001; "****" *P* ＜ 0.001. "ns" not significant. 95% CI: "Lower 95% CI of mean", "Upper 95% CI of mean".
